# Low Rate of SARS-CoV-2 Infections in Symptomatic Patients Attending a Pediatric Emergency Department

**DOI:** 10.3389/fped.2021.637167

**Published:** 2021-04-09

**Authors:** Christoph Zurl, Ernst Eber, Anna Siegl, Sabine Loeffler, Evelyn Stelzl, Harald H. Kessler, Markus Egger, Nina A. Schweintzger, Werner Zenz, Volker Strenger

**Affiliations:** ^1^Division of General Pediatrics, Department of Pediatrics and Adolescent Medicine, Medical University Graz, Graz, Austria; ^2^Section of Infectious Diseases and Tropical Medicine, Department of Internal Medicine, Medical University Graz, Graz, Austria; ^3^Division of Pediatric Pulmonology and Allergology, Department of Pediatrics and Adolescent Medicine, Medical University Graz, Graz, Austria; ^4^Emergency Department, Department of Pediatrics and Adolescent Medicine, Medical University Graz, Graz, Austria; ^5^Diagnostic & Research Institute of Hygiene, Microbiology and Environmental Medicine, Medical University Graz, Graz, Austria

**Keywords:** SARS-CoV-2, COVID - 19, epidemiology, respiratory infections, emergency department

## Abstract

Children and adolescents seem to be at lower risk of developing clinical symptoms of COVID-19. We analyzed the rate of SARS-CoV-2 infections among 3,605 symptomatic children and adolescents at 4,402 outpatient visits presenting to a pediatric emergency department. In a total of 1,105 (32.6%) episodes, the patients fulfilled clinical case definitions for SARS-CoV-2 infection and were tested by nucleic acid testing. A SARS-CoV-2 infection was diagnosed in 10/1,100 episodes (0.3% of analyzed episodes, 0.91% of validly tested patients). Symptoms at presentation did not differ between patients with and without SARS-CoV-2 infection, apart from the frequency of measured temperature ≥37.5°C at presentation. Three percent of analyzed children reported disturbances of olfactory or gustatory senses, but none of them was infected with SARS-CoV-2. The rate of SARS-CoV-2 infections among symptomatic children and adolescents was low and SARS-CoV-2 infections could not reliably be differentiated from other infections without nucleic acid testing.

## Introduction

While children and adolescents seem to be at lower risk of becoming infected with severe acute respiratory syndrome coronavirus 2 (SARS-CoV-2) and of developing clinical symptoms of coronavirus disease 2019 (COVID-19) (1, 2), they experience up to ten respiratory infections per year (3). Little is known about the epidemiology of COVID-19 in symptomatic children. Up to now, incidence of pediatric SARS-CoV-2 infections were mainly studied in relation to the number of all cases or to the general population (2, 4). We aimed to retrospectively analyse the rate of SARS-CoV-2 infections among symptomatic children and adolescents fulfilling clinical COVID-19 case definitions, which comprise respiratory symptoms, fever and sudden onset of anosmia, ageusia or dysgeusia.

## Methods

Similar to many other countries, restrictions for public life (including a partial lock down and closures of schools and day care centers) were imposed in Austria in mid-March 2020 and released stepwise from April onwards. Schools and day care facilities re-opened in May until July when holidays started. Access to health care facilities was restricted to acute illnesses and all health care institutions were obliged to identify patients with proven or suspected SARS-CoV-2 infections by screening systems (5–7). In our children's university hospital, serving a catchment area of about 150,000 children and adolescents, a screening questionnaire based on national definitions of suspected SARS-CoV-2 infections was implemented (5). These national definitions included clinical criteria (respiratory symptoms and fever) as well as epidemiological criteria (contact to a SARS-CoV-2 positive person or a recent stay in a region with SARS-CoV-2 community transmission). Since mid-April, ageusia or dysgeusia have been included in the case definition and SARS-CoV-2 community transmission is observed in our region. Symptoms and anamnestic details according to the national criteria for a suspected SARS-CoV-2 case were systematically surveyed by trained nursing staff prior to admission and are listed in Table 1. Any patient fulfilling at least one of the inquired items (i.e., fulfilling the national criteria for a suspected SARS-CoV-2 case) was further examined by a pediatrician to rule out or confirm SARS-CoV-2 infection. Diagnostic procedures (e.g., laboratory examinations, X-ray, sonography) were initiated at the discretion of the attending pediatrician. If no alternative diagnosis could be reliably established in the outpatient setting, nucleic acid testing from (naso- or oro-) pharyngeal swabs was performed. Swabs were collected by using the Copan ESwab™ collection system containing 1 mL of transport medium and stored at 2–8°C until transported to the Molecular Diagnostics Laboratory, Medical University of Graz. Samples were tested for SARS-CoV-2 RNA within 12 h of arrival. The presence of SARS-CoV-2 RNA was determined by real-time PCR (qPCR) using the *in vitro* diagnostics/Conformité Européenne (IVD/CE)-labeled Cobas®SARS-CoV-2 test (Roche Molecular Systems, Branchburg, NJ, USA) for use on the Cobas® 6800/8800 system (Roche Molecular Diagnostics, Rotkreuz, Switzerland). Further management (admission, discharge, treatment) was based on the clinical condition and not on SARS-CoV-2 test results. Categorical values are presented as absolute numbers and percentage and continuous variables are presented as medians and interquartile ranges. Differences were analyzed using Fisher's Exact Test or Mann–Whitney-*U*-Test as appropriate. Seven-day incidence rates were calculated as number of cases in Austria per week per 100,000 residents. These data are publically available from www.data.gv.at. Statistical analyses were performed using R 3.5.1 (www.r-project.org). Analysis of these data has been approved by the local ethics committee (32-636 ex 19/20).

**Table 1:** Characteristics of patients and items evaluated with the screening questionnaire based on national clinical case definitions for suspected SARS-CoV-2 infections and the frequencies of these criteria in pediatric patients with or without SARS-CoV-2 infection.

	**Episodes with positive SARS-CoV-2 PCR (*n* = 10)**	**Episodes with negative SARS-CoV-2 PCR (*n* = 1,090)**	
**Median age (IQR), years**	8.44 (1.24–11.97)	3.18 (1.18–7.42)	n.s.
**Male (%)**	5 (50%)	568 (52.1%)	n.s.
**Items inquired at screening:**			
Sore throat	2/10 (20%)	227/1033 (22%)	n.s.
Respiratory signs and symptoms	4/10 (40%)	458/1090 (42%)	n.s.
Laryngitis, hoarseness, stridor	1/9 (11.1%)	119/1042 (11.4%)	n.s.
Cough	4/10 (40%)	357/1042 (34.3%)	n.s.
Bronchitis, ronchi	1/10 (10%)	88/1030 (8.5%)	n.s.
Dyspnoe, shortness of breath	0/10 (0%)	113/1040 (10.9%)	n.s.
Tachypnoe (age adapted)	0/10 (0%)	66/1026 (6.4%)	n.s.
Temperature ≥37.5°C	7/10 (70%)	793/1090 (72.8%)	n.s.
reported prior to admission	6/10 (60%)	775/1018 (76.1%)	n.s.
Measured at admission	5/10 (50%)	205/1056 (19.4%)	p=0.03
Median temperature at screening (IQR), °C	37.9 (36.4–38.5)	36.8 (36.4–37.5)	n.s.
Sudden onset of anosmia or a-/dysgeusia	0/9 (0%)	16/619 (2.6%)	n.s.
Known contact to a confirmed case	0/10 (0%)	14/980 (1.4%)	n.s.
**Hospital admission**	5 (50%)	354/1090 (32.4%)	n.s.
**Clinical diagnoses** (SARS-CoV-2 results pending)			
Fever without source	5 (50%)	291 (26.7%)	n.s.
Respiratory infection	3 (30%)	193 (17.7%)	n.s.
Tonsillopharyngitis	1 (10%)	144 (13.2%)	n.s.
Acute gastrointestinal disease	0	121 (11.1%)	n.s.
Bronchitis	1 (10%)	48 (4.4%)	n.s.
Allergic reaction	0	5 (0.5%)	n.s.
Bronchial asthma	0	13 (1.2%)	n.s.
Dermatological disease	0	25 (2.3%)	n.s.
Infectious mononucleosis	0	11 (1.0%)	n.s.
Neurological disease	0	20 (1.8%)	n.s.
Pneumonia	0	18 (1.7%)	n.s.
Sepsis	0	11 (1.0%)	n.s.
Urinary tract infection	0	38 (3.5%)	n.s.
Otitis media	0	19 (1.7%)	n.s.
Other	0	133 (12.2%)	n.s.

*Some items were missing in some patients. Anosmia and a-/dysgeusia were not inquired before May 2020*.

## Results

**Figure 1:**
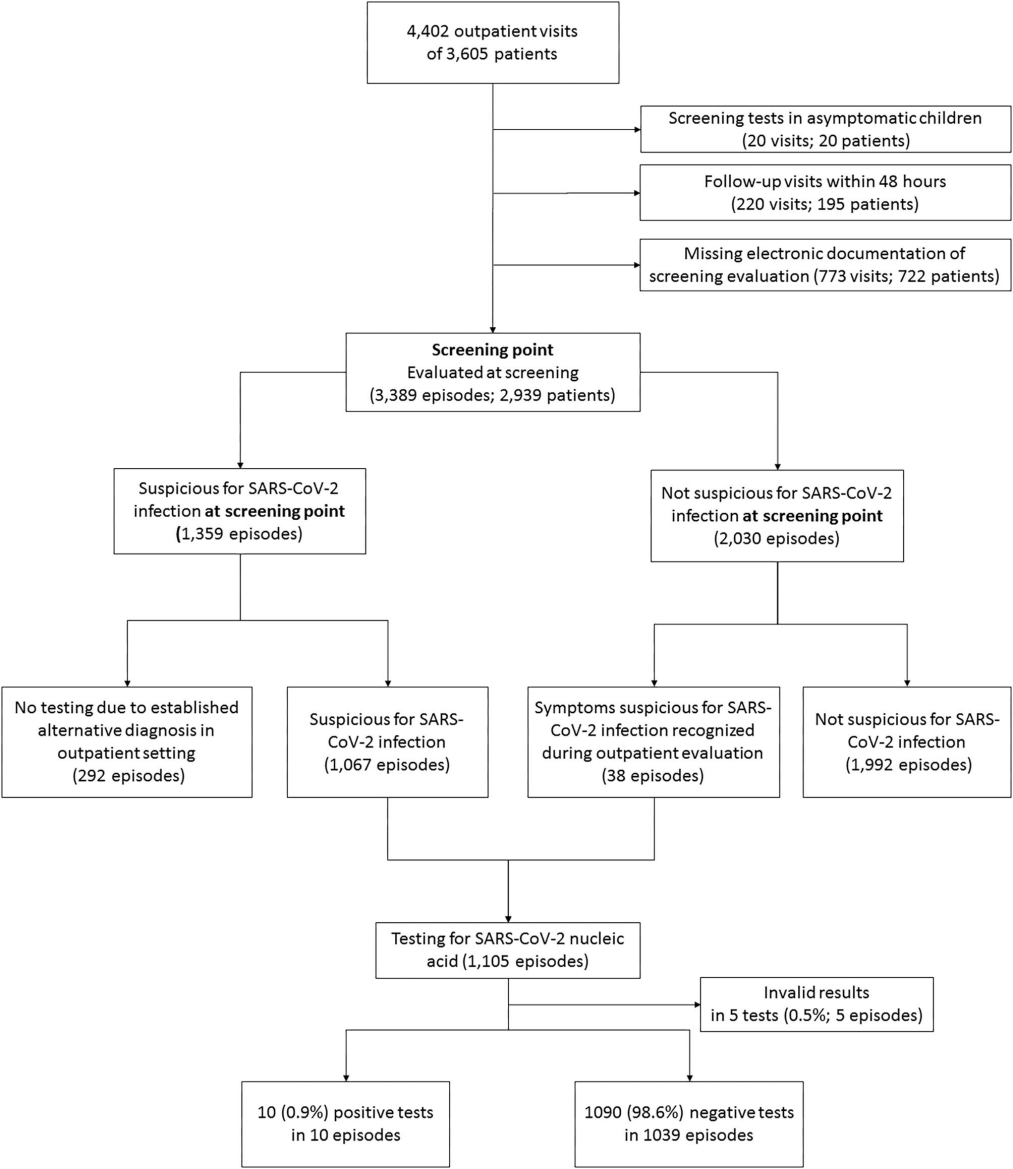
Flow chart of excluded and analyzed patients. A total of 595 of 3,605 patients (16.5%) had more than one visit. The range of visits per patient was 1 to 10.

**Figure 2:**
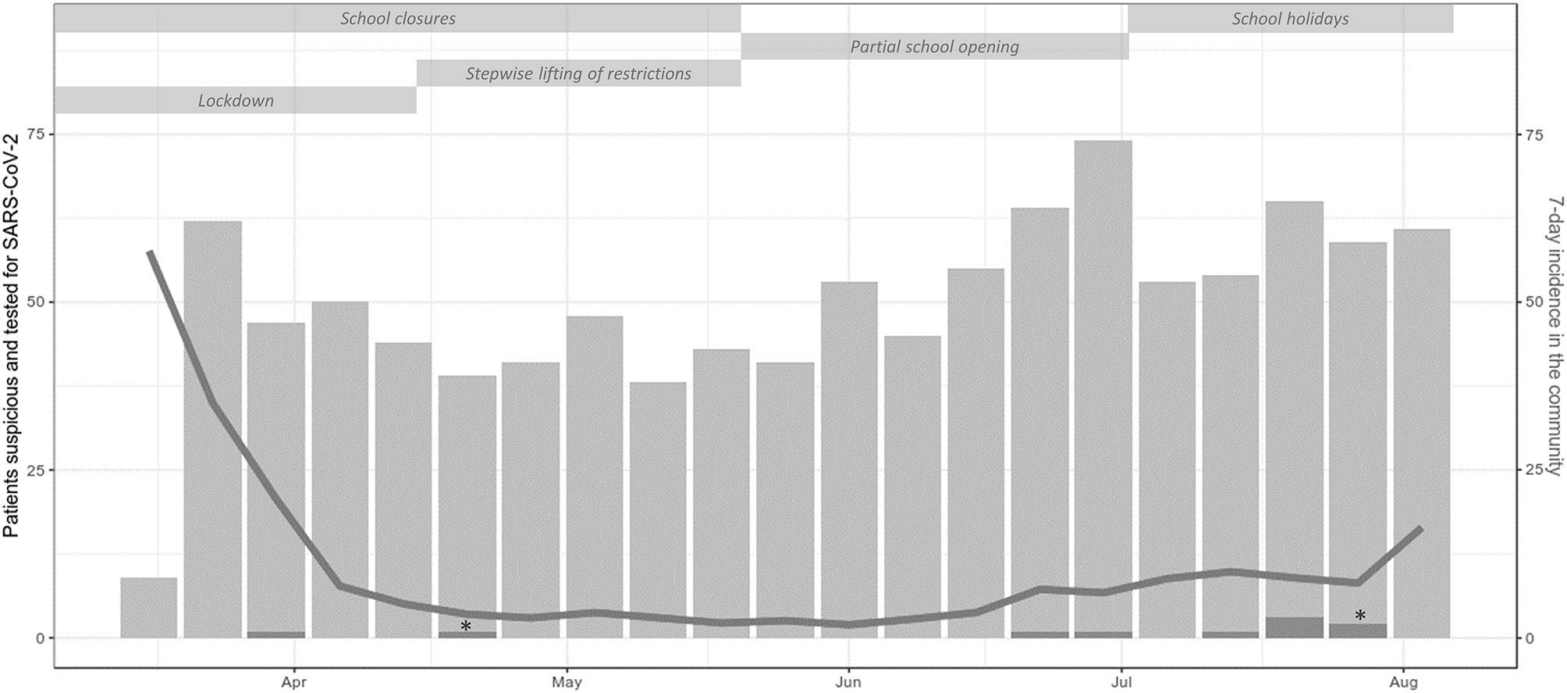
Counts per week of outpatient visits of patients suspicious and tested for SARS-CoV-2 infection (light gray bars) and SARS-CoV-2 positive patients (dark gray bars) including single patients with PIMS-TS (*), 7-day incidence in the community (gray line) and non-pharmaceutical measures during the study period. In the first week, screening for criteria suspicious for SARS-CoV-2 infection started on Thursday explaining the low number of tested patients in this week.

From March 19th to August 15th, 3,605 patients were examined at 4,402 outpatient visits. After exclusion of 1,013 visits of 859 patients (Figure 1), 3,389 visits (episodes) of 2,939 patients were further analyzed. At screening, symptoms suspicious for SARS-CoV-2 infection were reported in 1,359 episodes (40.1%). An alternative diagnosis was established by a pediatrician in 292 of these episodes. In 38 episodes, patients were initially considered not being suspicious for SARS-CoV-2 infection at screening but presented suspicious symptoms afterwards. Thus, in a total of 1,105 (32.6%) episodes, the patients were considered suspicious for SARS-CoV-2 infection and were tested by nucleic acid testing. After exclusion of five episodes with invalid tests, a SARS-CoV-2 infection was diagnosed in 10/1,100 (0.3% of analyzed episodes, 0.91% of validly tested patients). Two of them were diagnosed with pediatric inflammatory multi-system syndrome temporally associated with SARS-CoV-2 (PIMS-TS). Positivity rate per week varied depending on community transmission and ranged from 0 to 4% (Figure 2) and tended to be lower in children below 10 years with a positive result in 6/890 valid tests (0.67%) compared to children aged 10 years and older (4/210, 1.9%, not significant). During the observation period, the median overall 7-day incidence (including all age groups) was 7.0 cases per 100,000 inhabitants (range 1.9-57.6).

Symptoms and final clinical diagnoses are listed in Table 1. There were no statistically significant differences in the rates of symptoms between positively and negatively tested patients, apart from the frequency of measured temperature ≥37.5°C at presentation. In 742/997 (74.4%) visits temperature ≥37.5°C was only reported but not confirmed at presentation. The rate of patients showing both temperature ≥37.5°C and respiratory symptoms were similar for SARS-CoV-2 positive (20.0%) and SARS-CoV-2 negative patients (21.4%).

Anosmia, ageusia or dysgeusia were inquired in 628 episodes with SARS-CoV-2 testing and were reported in 16 (2.5%), but all of these patients were tested negative for SARS-CoV-2.

## Discussion

While a high proportion (>40%) of children presenting to the emergency department had symptoms suspicious for SARS-CoV-2 infection, we observed a very low positivity rate of only 0.91% in symptomatic children assessed systematically for signs and symptoms compatible with SARS-CoV-2 infection. This low rate of positive cases was observed in a setting with a moderate incidence of SARS-CoV-2 infections as well as a moderate incidence of other respiratory infections (decreased incidence of other respiratory infections during lockdown and reopening in spring and summer) and obviously positivity rate is influenced by these two factors. Similar results were obtained in an observational study from emergency departments in the UK where also only 1.2% of children with suspected COVID-19 had SARS-CoV-2 detected while positivity rate in adults with suspected COVID-19 was much higher (31.2%) (8). Higher positivity rates up to 6% depending on the study site were observed in the United States although also adolescents and young adults (up to 25 years) were included (9). As in other countries, presentations to our emergency department decreased notably compared to previous years as people might have avoided hospitals due to fear of transmission and an overall reduction of circulating infectious diseases (10, 11). With 40.1% of presenting children being suspicious for COVID-19 at initial screening, there was still a high proportion of children with signs and symptoms of infectious diseases. Based on the setting of a pediatric emergency department, further diagnostic work up for other infectious agents (like other respiratory viruses) was not routinely performed and therefore this was not included in our analyses. In our cohort, symptoms at presentation did not differ between patients with and without SARS-CoV-2 infection, although interpretation is limited as only a low number of patients were tested positive. Anosmia, ageusia and dysgeusia were reported several times in SARS-CoV-2 negative but in none of the SARS-CoV-2 positive patients, although these symptoms are reported to be rather specific for SARS-CoV-2 infection in adults.

In conclusion, the rate of SARS-CoV-2 infections among pediatric patients fulfilling the clinical case definitions was very low and SARS-CoV-2 infections could not reliably be differentiated from other infections without nucleic acid testing.

## Data Availability Statement

The raw data supporting the conclusions of this article will be made available by the authors, without undue reservation.

## Ethics Statement

The studies involving human participants were reviewed and approved by Ethics Committee of the Medical University Graz. Written informed consent from the participants' legal guardian/next of kin was not required to participate in this study in accordance with the national legislation and the institutional requirements.

## Author Contributions

CZ and VS conceptualized and designed the study, carried out the initial analyses, drafted the initial manuscript, and reviewed and revised the manuscript. EE and WZ critically analyzed and interpreted data and reviewed and revised the manuscript. NS carried out the initial analyses and reviewed and revised the manuscript. AS, SL, ME, ES, and HK designed the data collection instruments, collected data, reviewed, and revised the manuscript. All authors read and approved the final version of the manuscript.

## Conflict of Interest

The authors declare that the research was conducted in the absence of any commercial or financial relationships that could be construed as a potential conflict of interest.
